# CircHIPK3 promotes the progression of B-cell acute lymphoblastic leukemia in children by binding to STAT3

**DOI:** 10.3389/fphar.2026.1884978

**Published:** 2026-09-17

**Authors:** Yu Zheng, Yunpeng Li, Xiuli Zhu, Yan Chen, Yuqiao Diao

**Affiliations:** Department of Pediatrics, The Fourth Hospital of Hebei Medical University, Shijiazhuang, Hebei, China

**Keywords:** B-cell acute lymphoblastic leukemia, CircHIPK3, leukemic infiltration, STAT3, treatment

## Abstract

**Background:**

Circular RNAs (circRNAs) have emerged as critical post-transcriptional regulators in hematological malignancies; however, the specific role and molecular mechanism of circHIPK3 in pediatric B-cell acute lymphoblastic leukemia (B-ALL) remain unexplored.

**Methods:**

circHIPK3 expression was profiled in clinical B-ALL specimens and cell lines. The biological functions were evaluated using CCK-8, flow cytometry, and *in vivo* NOD/SCID xenograft models for leukemic infiltration assessment. Mechanistic interactions were deciphered via RNA immunoprecipitation (RIP), RNA pull-down, and ubiquitination assays.

**Results:**

CircHIPK3 was aberrantly upregulated in B-ALL tissues and cells, exhibiting exceptional stability and predominant cytoplasmic localization. CircHIPK3 silencing significantly impaired leukemic cell proliferation, induced G0/G1 cell cycle arrest, and triggered apoptosis. Mechanistically, we identified a direct physical association between cytoplasmic circHIPK3 and STAT3. CircHIPK3 did not alter STAT3 transcription but rather acted as a protein scaffold that shielded STAT3 from ubiquitin-mediated proteasomal degradation, thereby enhancing its protein stability. Ectopic STAT3 expression reversed the tumor-suppressive phenotypes caused by circHIPK3 deficiency. Consistently, circHIPK3 knockdown attenuated leukemic infiltration in xenograft mice.

**Conclusion:**

Our findings characterize circHIPK3 as a potent oncogenic driver in B-ALL that functions by stabilizing STAT3 via the ubiquitin-proteasome pathway. Targeting the circHIPK3/STAT3 axis may represent a potential therapeutic strategy for B-ALL treatment.

## 1 Introduction

B-cell acute lymphoblastic leukemia (B-ALL) stands as the predominant hematological malignancy diagnosed in the pediatric population, originating from the malignant transformation of B-cell precursors (Inaba and Mullighan, 2020). Despite remarkable strides in risk-stratified chemotherapy and the advent of immunotherapies like CAR-T cells, which have elevated the 5-year overall survival rate to nearly 90%, the prognosis for patients with refractory or relapsed disease remains poor (Zhang et al., 2016; Gaudichon et al., 2019). Relapse often stems from clonal evolution and the persistence of leukemia-initiating cells driven by complex, often elusive, molecular dysregulations (Tran and Hunger, 2022; Stubbs et al., 2015). Consequently, unraveling the intricate molecular networks governing B-ALL proliferation and survival is imperative for identifying therapeutic vulnerabilities and improving clinical outcomes.

In the landscape of non-coding RNAs, circular RNAs (circRNAs) have emerged as pivotal regulators of tumorigenesis. Characterized by a covalently closed loop structure lacking 5′ caps and 3′ poly-A tails, circRNAs exhibit exceptional resistance to exonuclease digestion, making them highly stable biomarkers (Kristensen et al., 2022; Chen and Shan, 2021). While initial studies primarily established circRNAs as “sponges” for microRNAs (miRNAs), recent evidence over the past decade has unveiled a more sophisticated mode of action: circRNAs can interact directly with RNA-binding proteins (RBPs) to modulate their stability, localization, or function (Han et al., 2025; Jia et al., 2021; Liu et al., 2022; Grelloni et al., 2024). Among these, circHIPK3 (derived from the HIPK3 gene) has been identified as an oncogenic driver in various solid tumors, such as gastric cancer and lung cancer (Siedlecki et al., 2024; Shang et al., 2023). Okholm et al. recently demonstrated that circHIPK3 is present at low copy number in bladder cancer cells and functions by nucleating multiple copies of IGF2BP2, potentially via phase separation, to regulate target mRNA stability. This finding highlights that circHIPK3 can exert regulatory functions through mechanisms that do not require 1:1 stoichiometric binding to its partners (Okholm et al., 2024). Tretti Parenzan et al. recently reported that circHIPK3, together with circZNF609, is among the few circRNAs previously associated with oncogenic behavior in ALL (Tretti Parenzan et al., 2024). Importantly, Gaffo et al. demonstrated that circHIPK3 exhibits striking and generalized upregulated expression in pediatric B-precursor acute lymphoblastic leukemia, further supporting its potential relevance in this disease (Gaffo et al., 2019). However, the expression profile and specific biological function of circHIPK3 in B-ALL remain largely under-investigated. More importantly, whether circHIPK3 can regulate key leukemogenic signaling pathways, such as the Signal Transducer and Activator of Transcription 3 (STAT3) axis—a pathway frequently hyperactivated in leukemia (Gasparoli et al., 2024; Ye et al., 2024)—via protein-protein interactions rather than canonical miRNA sponging, represents a significant gap in current knowledge.

In the present study, we sought to delineate the functional role and mechanistic underpinnings of circHIPK3 in B-ALL. We demonstrate that circHIPK3 is aberrantly upregulated in B-ALL clinical specimens and cell lines, correlating with aggressive clinicopathological features. Through a combination of *in vitro* and *in vivo* assays, we reveal that circHIPK3 is essential for leukemic cell proliferation and survival. Mechanistically, circHIPK3 acts as a molecular scaffold in the cytoplasm, associating to STAT3 and preventing its ubiquitin-mediated proteasomal degradation, thereby sustaining oncogenic STAT3 signaling. These findings highlight the circHIPK3/STAT3 axis as a promising therapeutic target for B-ALL treatment.

## 2 Materials and methods

### 2.1 Clinical specimens

Bone marrow (BM) aspirates were obtained from 30 pediatric patients diagnosed with B-cell acute lymphoblastic leukemia (B-ALL) and 30 healthy donors (serving as controls). The healthy donors were carefully age- and sex-matched to the B-ALL cohort. The healthy controls consisted of children who had undergone orthopedic surgery and had no history of hematologic or non-hematological malignancies, no history of active infectious diseases (such as HIV, hepatitis B, or hepatitis C), no autoimmune diseases, and who had not received any systemic immunomodulatory therapy. Healthy donors must have normal preoperative complete blood counts (CBC) and coagulation parameters, and sufficient bone or bone marrow tissue must be available for disposal during the procedure. Mononuclear cells (BMMCs) were isolated using density gradient centrifugation. All guardians of the participants provided written informed consent. Clinical parameters of the 30 pediatric B-ALL patients were retrospectively collected from electronic medical records, including age at diagnosis, sex, initial white blood cell (WBC) count, risk stratification. The study protocol was reviewed and approved by the Ethics Committee of The Fourth Hospital of Hebei Medical University (Approval No.: 2025KS042).

### 2.2 Cell culture and transfection

Human B-ALL cell lines (Reh, RS4; 11, and Nalm-6) were procured from ATCC. Peripheral blood mononuclear cells (PBMCs) were isolated from healthy volunteers. All cells were maintained in RPMI-1640 medium supplemented with 10% fetal bovine serum (FBS) and 1% penicillin/streptomycin at 37 °C in a humidified atmosphere containing 5% CO_2_. For gene silencing, small interfering RNA (siRNA) or short hairpin RNA (shRNA) targeting the junction site of circHIPK3 (si-circHIPK3 or sh-circHIPK3) and a non-targeting negative control (si-NC or sh-NC), as well as STAT3 overexpression plasmid (OE-STAT3) were synthesized by Gene-Chem (Shanghai, China). The sequence of siRNA and shRNA is listed in Supplementary Table S1. Transfection into Reh and Nalm-6 cells was performed using Lipofectamine 3000 reagent (Invitrogen) following the manufacturer’s instructions.

### 2.3 Nucleocytoplasmic fractionation

To determine the subcellular localization of circHIPK3, cytoplasmic and nuclear RNA fractions were isolated using the PARIS™ Kit (Ambion). GAPDH and U6 snRNA were utilized as controls for the cytoplasmic and nuclear fractions, respectively. The abundance of target transcripts in each fraction was quantified via RT-qPCR.

### 2.4 CircRNA stability assays

For the RNase R resistance assay, total RNA (2 μg) was incubated with 3 U/μg of RNase R or mock vehicle for 15 min at 37 °C. For the Actinomycin D assay, cells were treated with 2 μg/mL Actinomycin D (Sigma-Aldrich) to inhibit *de novo* transcription. RNA was harvested at indicated time points (0, 6, 12, and 24 h) to measure the half-lives of circHIPK3 and linear HIPK3 mRNA. Half-lives were calculated using one-phase exponential decay fitting in GraphPad Prism.

### 2.5 RNA extraction and quantitative real-time PCR (RT-qPCR)

Total RNA was extracted using the TRIzol reagent (Invitrogen). Reverse transcription was carried out using the PrimeScript™ RT Reagent Kit (Takara). Quantitative PCR was subsequently performed on an ABI 7500 System using the SYBR Green PCR Master Mix. Divergent primers were designed specifically to amplify the back-splicing junction of circHIPK3, HIPK3 mRNA, STAT3 mRNA (Supplementary Table S1). The relative expression levels were calculated, with GAPDH serving as the internal reference.

### 2.6 Cell proliferation assays

Cell viability was monitored using the Cell Counting Kit-8 (CCK-8, Dojindo). Transfected cells were seeded into 96-well plates. At 0, 24, 48, 72, and 96 h post-seeding, CCK-8 solution (10 μL) was added, and absorbance was measured at 450 nm after 2 h of incubation.

### 2.7 Cell cycle and apoptosis analysis

For cell cycle analysis, harvested cells were fixed in 70% ice-cold ethanol overnight at −20 °C. Subsequently, cells were stained with propidium iodide (PI) in the presence of RNase A (BD Biosciences) and analyzed using a flow cytometer (Beckman Coulter). The percentages of cells in G0/G1, S, and G2/M phases were determined using the Watson pragmatic model in FlowJo software.

For apoptosis assessment, cells were resuspended in binding buffer and double-stained with Annexin V-FITC and PI (BD Biosciences) for 15 min in the dark. The total apoptotic rate was calculated as the percentage of Annexin V-positive cells (including both early apoptotic, Annexin V^+^/PI^−^, and late apoptotic/necrotic, Annexin V^+^/PI^+^ populations) relative to the total cell count.

Flow cytometry gating strategy: For both assays, cells were first gated on FSC-A vs. SSC-A to exclude debris, followed by FSC-A vs. FSC-H gating to exclude doublets and select single cells. For apoptosis analysis, quadrants were set based on unstained and single-stained controls. All flow cytometry data were acquired on a Beckman Coulter flow cytometer and analyzed using FlowJo software (BD Biosciences).

### 2.8 Fluorescence *in situ* hybridization (FISH) and immunofluorescence (IF)

Specific Cy3-labeled probes targeting the circHIPK3 junction sequence (Supplementary Table S1) were designed by GenePharma. Cells were fixed, permeabilized, and hybridized with the probes overnight. For co-localization studies, the slides were incubated with an anti-STAT3 antibody (1:1,000, CST, #9139) followed by an FITC-conjugated secondary antibody. Nuclei were counterstained with DAPI. Images were captured using a confocal laser scanning microscope (Leica).

### 2.9 RNA immunoprecipitation (RIP)

RIP assays were conducted using the Magna RIP™ RNA-Binding Protein Immunoprecipitation Kit (Millipore). Cell lysates were incubated with magnetic beads conjugated with either anti-STAT3 antibody or control IgG antibody overnight at 4 °C. The co-precipitated RNAs were purified and subjected to RT-qPCR to quantify circHIPK3 enrichment.

### 2.10 RNA pull-down assay

Biotinylated probes targeting the circHIPK3 junction site and control probes (Supplementary Table S1) were incubated with streptavidin-coated magnetic beads (Invitrogen). The probe-bead complexes were then mixed with cell lysates overnight. The proteins pulled down by the probes were eluted and analyzed by Western blotting using anti-STAT3 antibodies (CST, #9139).

### 2.11 Protein stability and ubiquitination assays

To assess protein stability, cells were treated with the protein synthesis inhibitor Cycloheximide (CHX, 100 μg/mL) for 0, 3, 6, 9, and 12 h. STAT3 protein levels were monitored by Western blotting. For the ubiquitination assay, cells were treated with the proteasome inhibitor MG132 (10 μM) or the lysosomal inhibitor chloroquine (CQ; 50 μM, Sigma-Aldrich) for 12 h. Cell lysates were immunoprecipitated with an anti-STAT3 antibody (CST, #9139), and the ubiquitination levels were visualized by immunoblotting with an anti-Ubiquitin antibody (CST, #3936).

### 2.12 Western blot assays

Cells were lysed in RIPA buffer (Beyotime, P0013B) supplemented with protease and phosphatase inhibitors (Roche), and protein concentrations were determined using the BCA assay (Beyotime). Equal amounts of protein were separated by 10% SDS-PAGE and transferred onto PVDF membranes (Millipore). Membranes were blocked with 5% non-fat milk in TBST for 1 h at room temperature, then incubated with primary antibodies overnight at 4 °C. After washing, membranes were incubated with HRP-conjugated secondary antibodies for 1 h at room temperature. Protein bands were visualized using enhanced chemiluminescence (ECL, Millipore) and imaged on a ChemiDoc system (Bio-Rad). Band intensities were quantified using ImageJ software (NIH) and normalized to GAPDH. Primary antibodies used included anti-STAT3 (CST, #9139), anti-pSTAT3 (Tyr705) (CST, #9145), anti-BCL-2 (CST, #4223), anti-c-Myc (CST, #5605), and anti-GAPDH (CST, #5174), all at 1:1,000 dilution. HRP-conjugated secondary antibodies (CST, #7074 and #7076) were used at 1:2,000 dilution.

### 2.13 *In vivo* xenograft models

Animal experiments were approved by the Animal Care and Use Committee of The Fourth Hospital of Hebei Medical University. All methods were carried out in accordance with relevant guidelines and regulations and in compliance with the ARRIVE guidelines 2.0. Ten male NOD/SCID mice (4–5 weeks old) were randomly allocated to the sh-NC or sh-circHIPK3 groups using a computer-generated random number sequence (n = 5 per group). Male NOD/SCID mice were intravenously injected via the tail vein with Nalm-6 cells stably transfected with sh-NC or sh-circHIPK3. The investigator responsible for cell injection, tumor monitoring, and data analysis was blinded to the group allocation. Upon sacrifice, spleens were harvested, photographed, and weighed.

### 2.14 Analysis of leukemic infiltration

Single-cell suspensions were prepared from the bone marrow and spleens of the xenograft mice. Cells were stained with anti-human CD45 and anti-human CD19 antibodies (BioLegend, #304012, #302219) to distinguish human leukemic cells from murine cells. The infiltration rate was quantified by flow cytometry.

### 2.15 Histological and immunohistochemical (IHC) analysis

Spleen tissues were fixed in 4% paraformaldehyde, embedded in paraffin, and sectioned. Hematoxylin and eosin (H&E) staining was performed for histological observation. For IHC, sections were incubated with anti-STAT3 antibodies. Images were acquired using a light microscope.

### 2.16 Statistical analysis

All experiments were repeated at least three times. Data are presented as mean ± standard deviation (SD). Statistical comparisons between two groups were performed using the Student’s t-test, while multi-group comparisons were analyzed using one-way ANOVA followed by Tukey’s *post hoc* test. For the CHX chase assay, data were analyzed using two-way repeated-measures ANOVA with factors, followed by Sidak’s *post hoc* test for multiple comparisons at each time point. For cell cycle distribution, comparisons between two groups (si-NC vs. si-circHIPK3) were performed using Student’s t-test for each phase (G0/G1, S, G2/M) independently. For rescue experiments involving three or more groups, one-way ANOVA followed by Tukey’s *post hoc* test was applied. A P-value < 0.05 was considered statistically significant. Data analysis was conducted using GraphPad Prism 8.0 software.

## 3 Results

### 3.1 Upregulation and molecular characterization of circHIPK3 in B-ALL

Initial investigation into the expression profile of circHIPK3 was conducted using clinical specimens to ascertain its potential involvement in B-ALL pathology. RT-qPCR analysis of bone marrow aspirates revealed that circHIPK3 abundance was aberrantly elevated in pediatric B-ALL patients relative to healthy donors (P < 0.001; Figure 1A). High circHIPK3 expression was significantly correlated with younger age (<10 years, P = 0.025), higher risk stratification (P = 0.041), and higher initial WBC counts (P = 0.035) (Table 1). This upregulation trend was recapitulated *in vitro*; compared to PBMCs from healthy individuals, B-ALL cell lines (including Reh, RS4; 11, and Nalm-6) exhibited significantly higher circHIPK3 levels, with Reh and Nalm-6 showing the most prominent increase (P < 0.001; Figure 1B). We then selected Reh and Nalm-6 as the primary model systems. These two cell lines represent the most common genetic subtypes of pediatric B-ALL—carrying ETV6-RUNX1 and ETV6-PDGFRB fusions, respectively—while RS4; 11, which harbors the MLL-AFF1 fusion characteristic of high-risk infant B-ALL.

**FIGURE 1:**
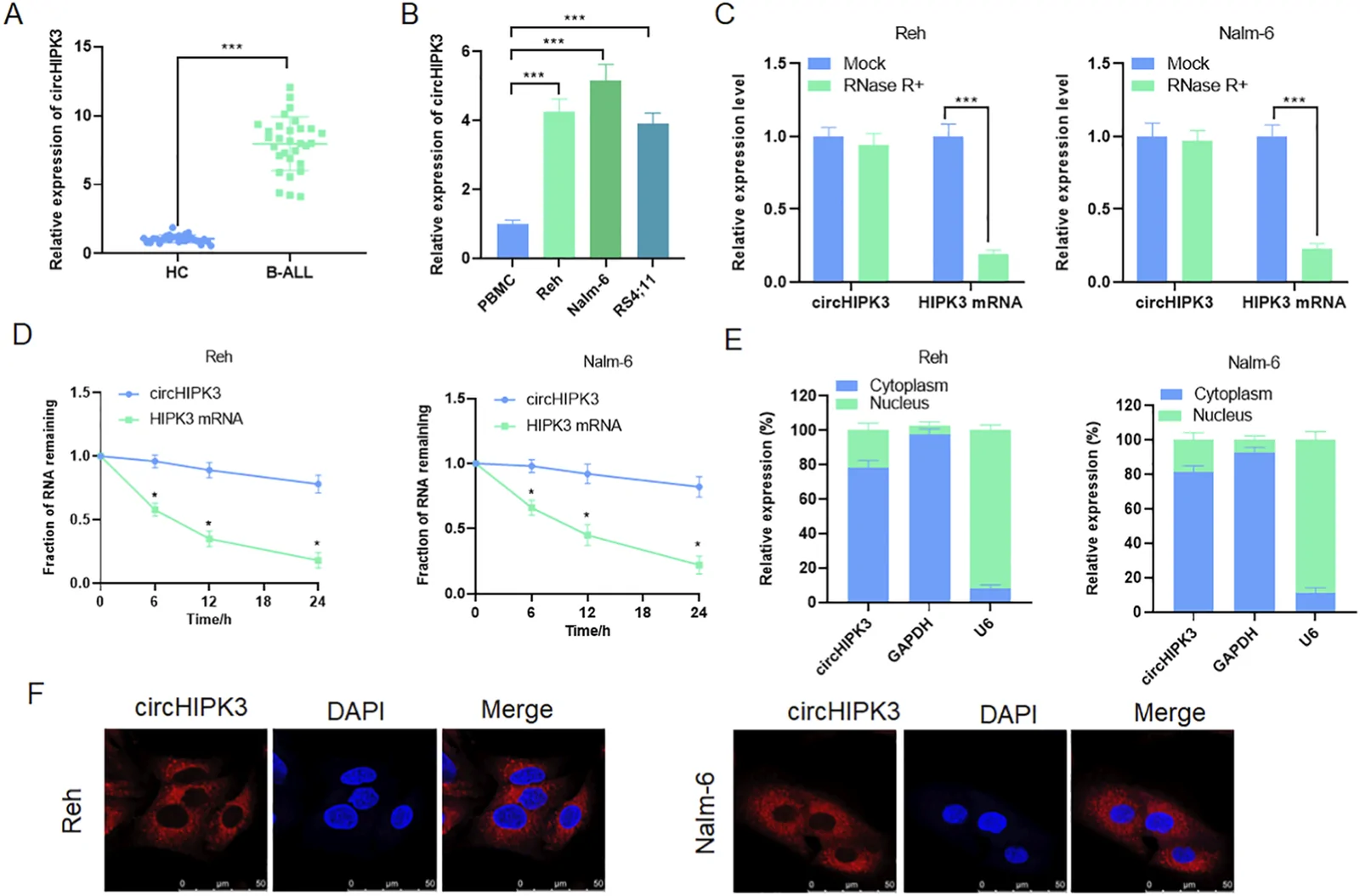
Aberrant upregulation and molecular characterization of circHIPK3 in B-ALL. **(A)** RT-qPCR analysis of circHIPK3 expression in bone marrow aspirates from pediatric B-ALL patients (n = 30) compared to healthy donors (n = 30). **(B)** Differential expression of circHIPK3 in B-ALL cell lines (Reh, RS4; 11, and Nalm-6) versus normal PBMC. **(C)** Characterization of circular structure via RNase R^+^ digestion. Total RNA from Reh and Nalm-6 cells was treated with RNase R^+^, followed by RT-qPCR detection of circHIPK3 and linear HIPK3 mRNA. **(D)** Transcript stability assessment. Cells were treated with Actinomycin D to inhibit transcription, and RNA half-lives were analyzed at indicated time points. **(E)** Nucleocytoplasmic fractionation analysis showing the relative abundance of circHIPK3 in the nuclear and cytoplasmic fractions. **(F)** Representative FISH images demonstrating the predominant cytoplasmic localization of circHIPK3 (red) in B-ALL cells. Nuclei were counterstained with DAPI (blue). Scale bar = 50 μm. *P < 0.05, ***P < 0.001.

**TABLE 1:** Correlation between circHIPK3 expression and clinicopathological characteristics in 30 B-ALL patients.

Clinicopathological	Total (n = 30)	circHIPK3 low (n = 15)	circHIPK3 high (n = 15)	P-value
Age (years)	​	​	​	0.025*
<10	20	7	13	​
≥10	10	8	2	​
Gender	​	​	​	0.462
Male	16	9	7	​
Female	14	6	8	​
Initial WBC count (×10^9^/L)	​	​	​	0.035*
<50	17	12	5	​
≥50	13	3	10	​
Risk stratification	​	​	​	0.041*
Standard risk	19	13	6	​
High risk	11	2	6	​

*P < 0.05 (Mann-Whitney U test or Kruskal-Wallis test).

To rigorously validate the circular characteristics of the identified transcript, we performed an RNase R digestion assay. As anticipated, while linear HIPK3 mRNA was largely depleted, circHIPK3 demonstrated substantial resistance to exonuclease digestion in both Reh and Nalm-6 cells, confirming its closed-loop structure (P < 0.001; Figure 1C). Furthermore, transcript stability was assessed by inhibiting *de novo* RNA synthesis with Actinomycin D. The results indicated that the half-life of circHIPK3 (>24 h) was significantly extended compared to linear HIPK3 mRNA (6.40 h for Reh, 9.33 for Nalm-6), implying superior stability (Figure 1D). Finally, to infer its functional mechanism, we determined the subcellular distribution of circHIPK3. Both nucleocytoplasmic fractionation assays and FISH provided converging evidence that circHIPK3 is predominantly enriched in the cytoplasm of B-ALL cells (Figures 1E,F), suggesting it may function via post-transcriptional regulation.

### 3.2 CircHIPK3 knockdown impairs proliferation and induces cell cycle arrest and apoptosis in B-ALL cells

To delineate the biological function of circHIPK3 in leukemogenesis, loss-of-function assays were performed by transfecting Reh and Nalm-6 cells with circHIPK3-specific siRNAs. RT-qPCR validation confirmed a marked reduction in circHIPK3 levels in the si-circHIPK3 group compared with the si-NC (P < 0.001), linear HIPK3 mRNA levels remained comparable to those in si-NC (P > 0.05), ensuring the efficacy of the knockdown (Figure 2A; Supplementary Figure S1). Subsequent functional evaluations unveiled an important role for circHIPK3 in sustaining leukemia cell growth. CCK-8 assays demonstrated that circHIPK3 silencing significantly attenuated the viability of both Reh and Nalm-6 cells over a 96 h period (P < 0.01; Figure 2B). This growth inhibition was mechanistically linked to cell cycle disruption; flow cytometry analysis revealed that circHIPK3 depletion halted cell cycle progression, characterized by a substantial accumulation of cells in the G0/G1 phase (P = 0.0032; P = 0.0055) and a concomitant decrease in the S-phase population (P = 0.0038; P = 0.0029) (Figure 2C). Furthermore, Annexin V/PI double-staining assays corroborated the tumor-suppressive effects of circHIPK3 knockdown, as evidenced by a sharp increase in the total apoptotic rate in si-circHIPK3-transfected cells relative to si-NC (P = 0.0006, P = 0.0011; Figure 2D).

**FIGURE 2:**
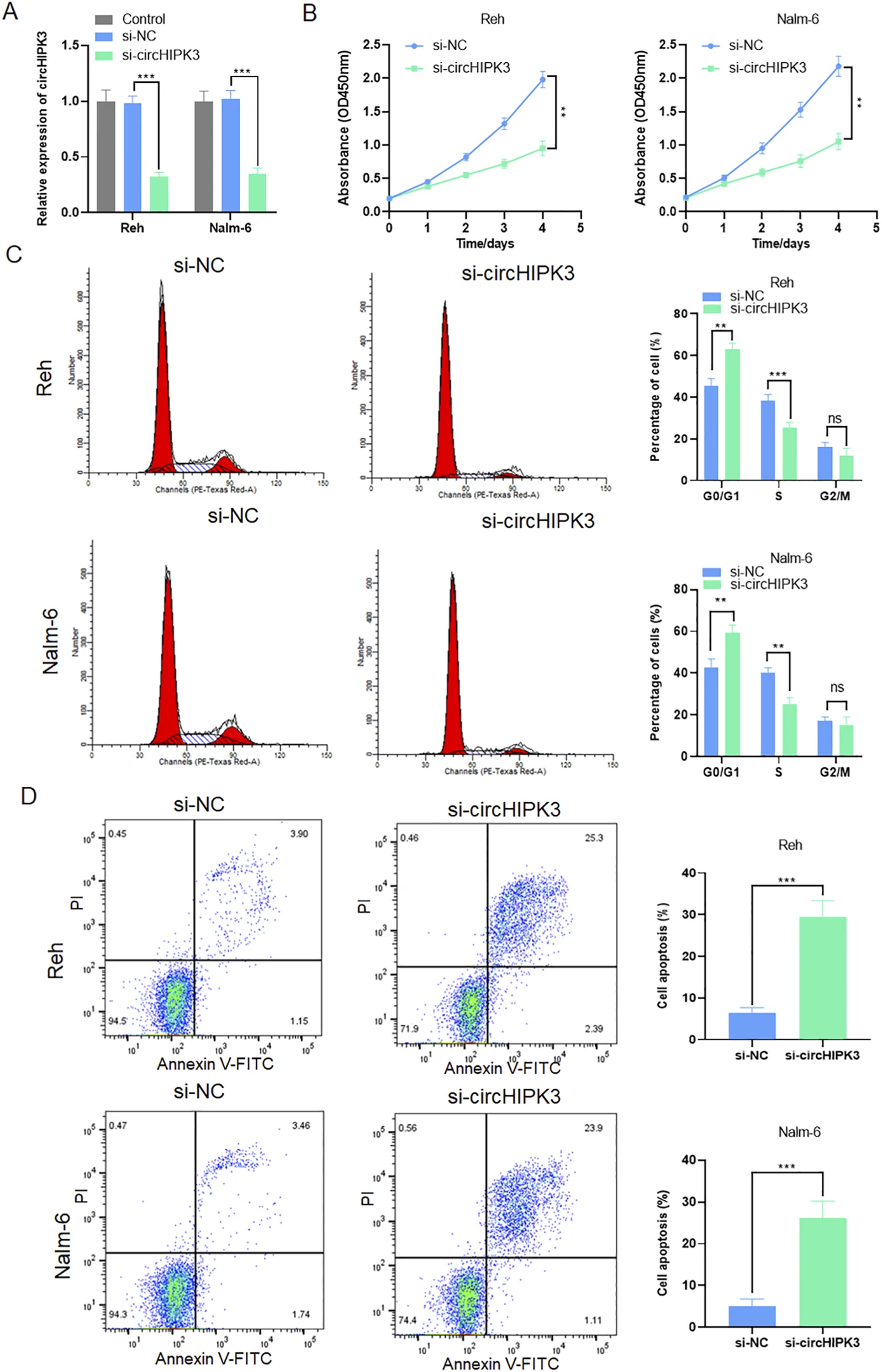
Depletion of circHIPK3 suppresses leukemic cell growth and induces cell cycle arrest and apoptosis. **(A)** CircHIPK3 levels in Reh and Nalm-6 cells transfected with si-NC or si-circHIPK3 were measured by RT-qPCR. **(B)** Assessment of cell viability using CCK-8 assays at 0, 1, 2, 3, and 4 days post-transfection. **(C)** Flow cytometry analysis of cell cycle distribution. The percentage of cells in G0/G1, S and G2/M phases was quantified by flow cytometry. **(D)** Flow cytometry evaluation of apoptosis rate via Annexin V-FITC/PI double staining. **P < 0.01, ***P < 0.001.

To rule out the transient nature and potential off-target effects of siRNA transfection, we have now performed shRNA-mediated stable knockdown of circHIPK3 (sh-circHIPK3) in both Reh and Nalm-6 lines. Consistent with our siRNA data, stable transfection of sh-circHIPK3 robustly decreased circHIPK3 levels (Supplementary Figure S2A). Furthermore, CCK-8 and flow cytometry assays confirmed that stable knockdown profoundly suppressed B-ALL cell proliferation and promoted apoptosis (Supplementary Figures S2B,C), perfectly mirroring the results obtained from transient siRNA experiments. These new data strongly solidify the reproducibility of our phenotypic observations. Collectively, these findings suggest that circHIPK3 is essential for B-ALL cell proliferation and survival.

### 3.3 High expression of STAT3 and its direct physical association with circHIPK3 in B-ALL cells

Given previous literature hinting at a regulatory interplay between circRNAs and STAT signaling, we hypothesized that circHIPK3 might function by directly binding to STAT3. First, we evaluated the expression status of STAT3 in clinical specimens. RT-qPCR analysis highlighted an aberrant upregulation of STAT3 mRNA in B-ALL samples compared to the control group (P < 0.001; Figure 3A). Confocal microscopy revealed a distinct spatial colocalization, where the fluorescent signals of circHIPK3 and STAT3 protein significantly overlapped within the cytoplasm of Reh and Nalm-6 cells (Figure 3B). To substantiate this physical association at the molecular level, RIP assays were performed. Endogenous circHIPK3 was enriched in the immune-precipitates pulled down by the anti-STAT3 antibody compared to the non-specific IgG control (P = 0.002; Figure 3C). This molecular affinity was further verified via biotinylated RNA pull-down assays. Western blot analysis of the retrieved complexes demonstrated that the biotin-labeled circHIPK3 probe successfully captured the STAT3 protein, whereas the negative control probe failed to show any detectable binding (Figure 3D). To predict the potential binding interface between circHIPK3 and STAT3, we performed molecular docking using the HDOCK server. The three-dimensional structure of circHIPK3 was modeled using 3dRNA with circular RNA option enabled, and the STAT3 crystal structure was retrieved from PDB (ID: 6TLC). The optimal docking model with the lowest energy score revealed that circHIPK3 intimately docks into a binding pocket within the SH2 domain of STAT3 (HDOCK score: 370.05; Confidence score: 0.9879) (Figure 3E). The interaction is robustly stabilized by multiple hydrogen bonds and electrostatic interactions. Specifically, key amino acid residues of STAT3, including Gln643, Leu645, Asn646, Glu652, Met660, Ile665, Lys685, Tyr686, Arg688 and Pro689, form strong hydrogen bonds with specific nucleotide bases of circHIPK3. This structural mapping provides molecular evidence for the direct and specific binding between circHIPK3 and STAT3. Collectively, these data provide evidence of a direct physical association between circHIPK3 and STAT3.

**FIGURE 3:**
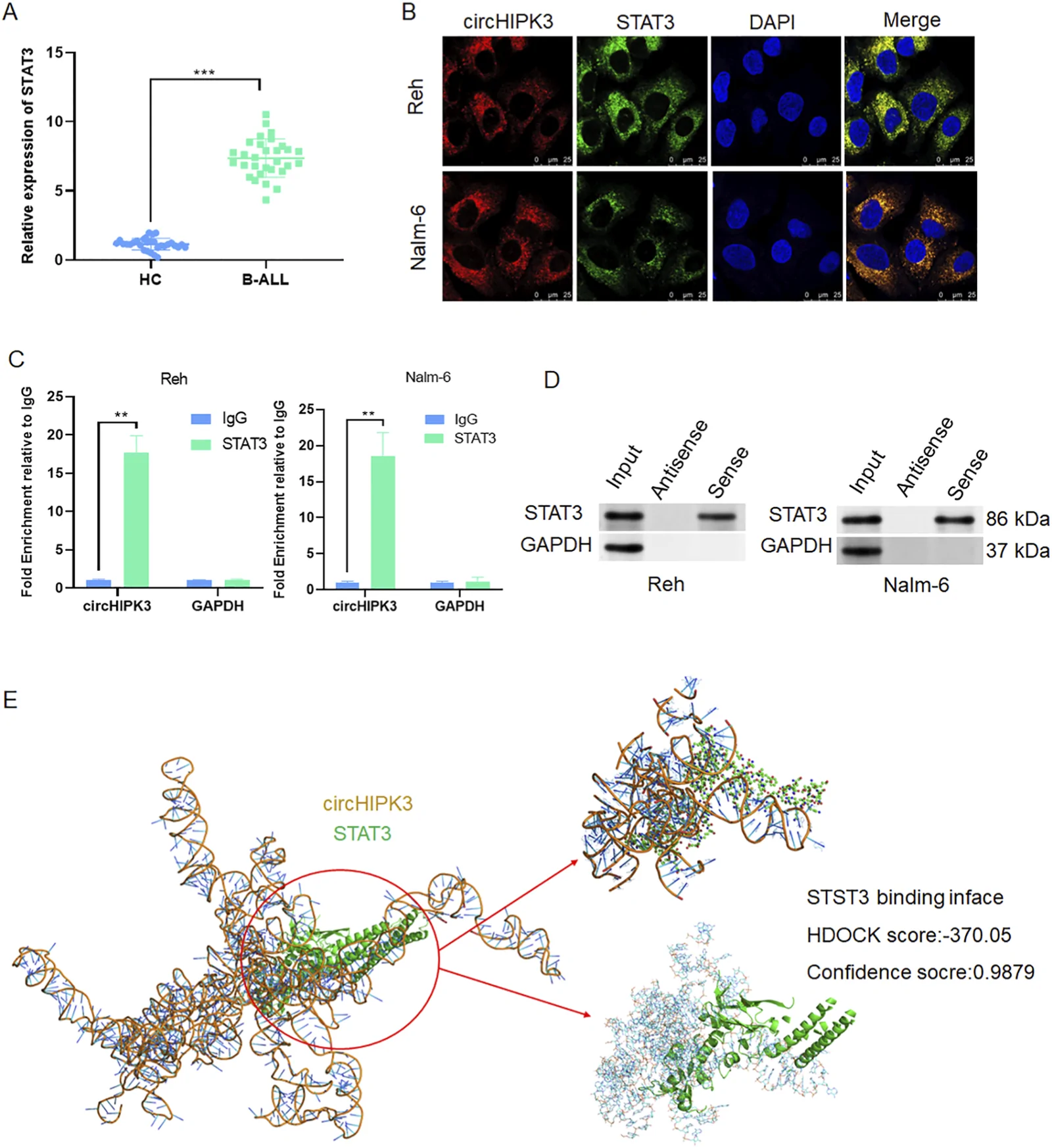
circHIPK3 physically interacts with STAT3 protein. **(A)** RT-qPCR quantification of STAT3 mRNA levels in clinical B-ALL bone marrow samples relative to healthy controls. **(B)** Confocal microscopy showing the spatial colocalization of circHIPK3 (FISH, red) and STAT3 protein (IF, green) in the cytoplasm of Reh and Nalm-6 cells. Scale bar = 25 μm. **(C)** RIP assay performed with anti-STAT3 antibody or IgG control. The enrichment of circHIPK3 was quantified by RT-qPCR. **(D)** RNA pull-down assay using biotinylated circHIPK3 probes or NC probes. STAT3 level was detected by Western blot **(E)** Graphical exhibition of molecular binding between circHIPK3 and STAT3 protein (PDB ID: 6TLC) using the HDOCK Server. **P < 0.01, ***P < 0.001.

### 3.4 circHIPK3 stabilizes STAT3 protein by antagonizing ubiquitin-proteasome degradation

To decipher the regulatory impact of the circHIPK3-STAT3 interaction, we assessed STAT3 expression following circHIPK3 silencing. Western blot analysis revealed a marked reduction in STAT3 protein levels in si-circHIPK3-transfected cells compared to controls (P < 0.001; Figure 4A). RT-qPCR data showed no significant fluctuation in STAT3 mRNA abundance (Figure 4B), implying that circHIPK3 modulates STAT3 at the post-transcriptional level rather than regulating its synthesis.

**FIGURE 4:**
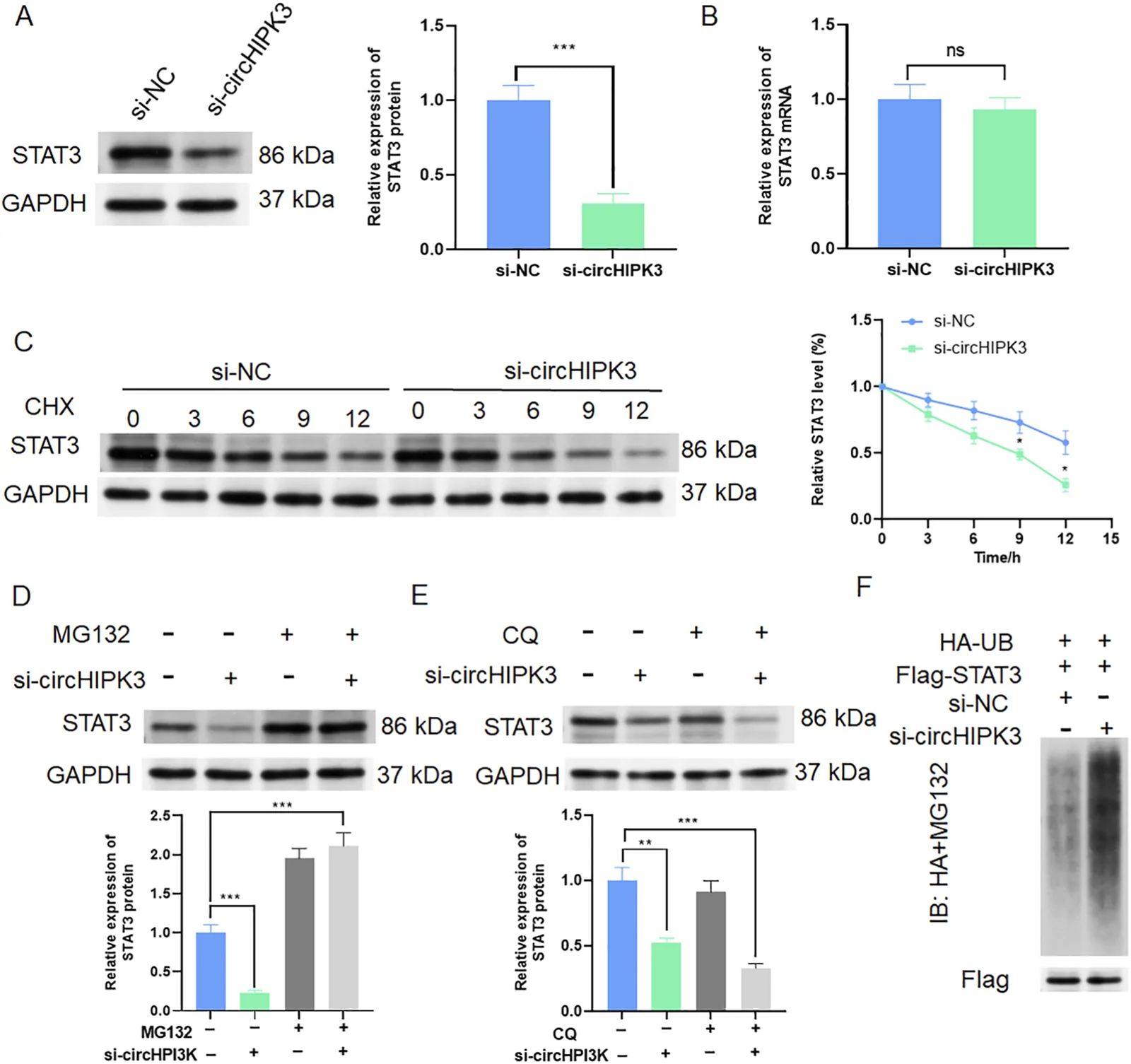
circHIPK3 stabilizes STAT3 by antagonizing ubiquitin-proteasome degradation. **(A)** Western blot analysis of STAT3 protein levels in Reh cells transfected with si-NC or si-circHIPK3. **(B)** RT-qPCR analysis of STAT3 mRNA expression in si-NC or si-circHIPK3 groups, showing no significant change at the transcriptional level. **(C)** Protein quantification in the CHX chase assay. Cells were treated with CHX (100 μg/mL) for the indicated times. The degradation rate of STAT3 protein was monitored by Western blotting. **(D,E)** Western blotting analysis STAT3 protein expression in circHIPK3 knockdown-cells after MG132 (10 μM) or CQ (50 μM) treatment. **(F)** Ubiquitination assay. Cells were treated with MG132 followed by immunoprecipitation with anti-STAT3 antibody. The ubiquitination levels were visualized using an anti-Ubiquitin antibody. *P < 0.05, ***P < 0.001.

Based on this discrepancy, we hypothesized that circHIPK3 might influence the stability of the STAT3 protein. A CHX chase assay was employed to monitor protein turnover. In control cells, STAT3 exhibited a relatively long half-life; however, circHIPK3 knockdown led to a markedly accelerated degradation rate of STAT3 (P = 0.0058; Figure 4C). To further pinpoint the degradation mechanism, we performed ubiquitination assays. The proteasome inhibitor MG132 rescued STAT3 protein expression in Reh cells upon circHIPK3 knockdown (Figure 4D). In contrast, inhibition of the lysosomal pathway by CQ failed to rescue STAT3 degradation, suggesting that circHIPK3 regulates STAT3 via a proteasome-dependent pathway (Figure 4E). Silencing circHIPK3 resulted in a substantial accumulation of poly-ubiquitinated STAT3 species (Figure 4F). These findings collectively suggest that circHIPK3 functions as a stabilizer, protecting STAT3 from ubiquitin-mediated proteasomal degradation.

### 3.5 STAT3 overexpression rescues the tumor-suppressive phenotypes induced by circHIPK3 deficiency

To conclusively determine whether the oncogenic function of circHIPK3 is mediated through the stabilization of STAT3, we performed rescue experiments by ectopically expressing STAT3 in circHIPK3-silenced cells. Western blot analysis first validated the experimental system, showing that the diminished STAT3 protein levels resulting from circHIPK3 knockdown were successfully restored upon transfection with the STAT3 overexpression plasmid (P < 0.001; Figure 5A). Subsequent functional assays demonstrated that the inhibition of cell proliferation, originally triggered by si-circHIPK3, was significantly mitigated in the presence of exogenous STAT3 (P < 0.001; Figure 5B). Similarly, the cell cycle arrest at the G0/G1 phase was alleviated, with the cell population distribution returning to near-baseline levels (P < 0.001; Figure 5C). Furthermore, the enhanced apoptosis rate induced by circHIPK3 silencing was largely abrogated by STAT3 overexpression (P < 0.001; Figure 5D). We next investigated whether this stabilization translates into sustained transcriptional activity of the STAT3 signaling pathway. Western blot analysis revealed that knockdown of circHIPK3 resulted in a profound downregulation of transcriptionally active phosphorylated STAT3 (p-STAT3), anti-apoptotic proteins (BCL-2) and the oncogenic transcription factor c-Myc; these downregulations were reversed by STAT3 overexpression (P < 0.001; Figure 5E). Taken together, these findings confirm that circHIPK3 exerts its pro-tumorigenic effects in B-ALL largely via the STAT3 signaling axis.

**FIGURE 5:**
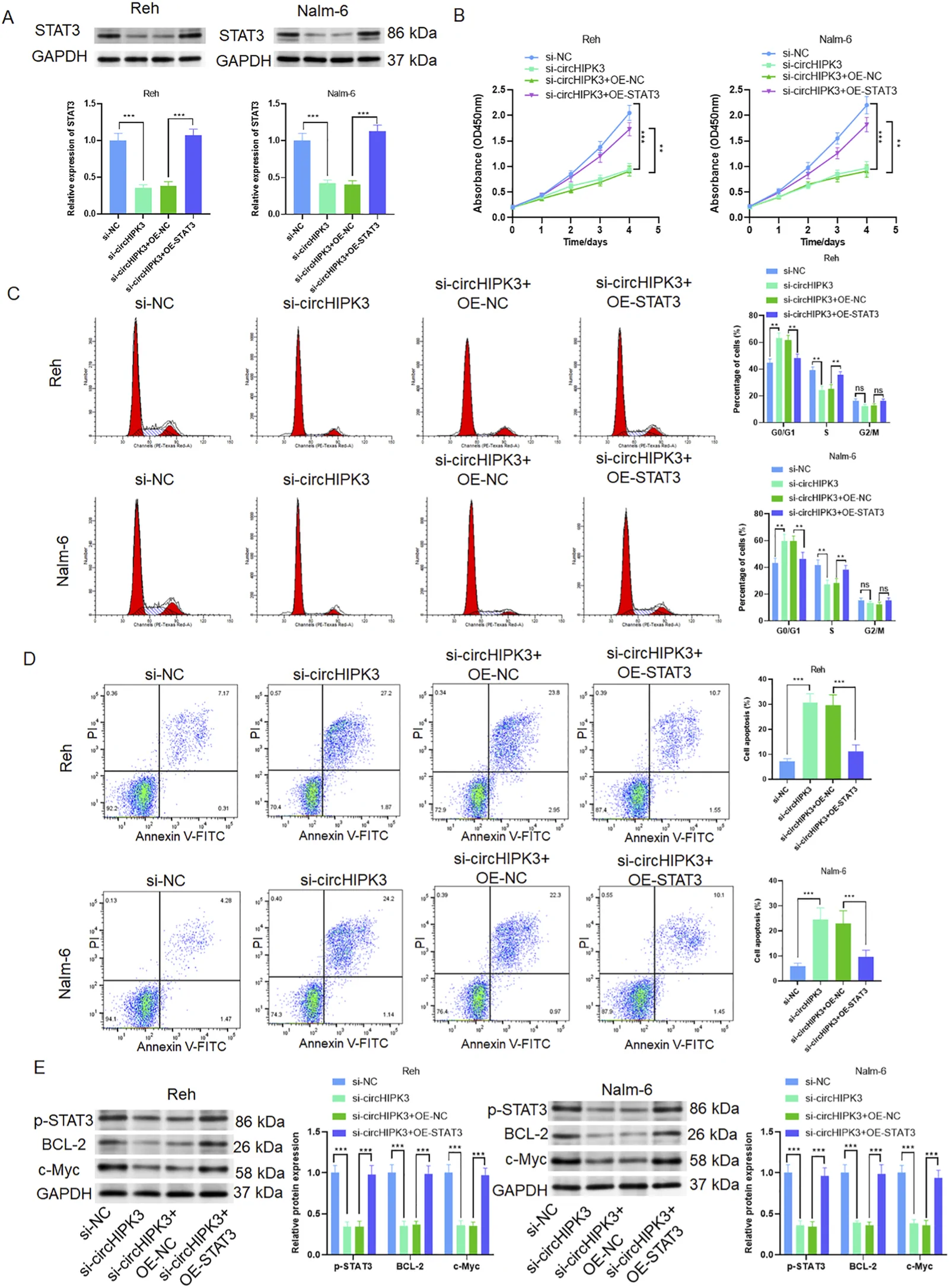
STAT3 overexpression rescues the tumor-suppressive phenotypes induced by circHIPK3 deficiency. **(A)** Western blot confirmation of STAT3 protein restoration in circHIPK3-silenced cells co-transfected with a STAT3 overexpression plasmid (OE-STAT3). **(B)** CCK-8 assays showing that STAT3 restoration mitigates the anti-proliferative effects of circHIPK3 knockdown. **(C)** Flow cytometry analysis cell cycle distribution, indicating the alleviation of G0/G1 arrest in the rescue group. **(D)** Flow cytometry analysis of apoptosis, demonstrating that ectopic STAT3 expression counteracts the apoptosis induced by circHIPK3 depletion. **(E)** Western blot confirmation of p-STAT3, BCL-2 and c-Myc protein restoration in circHIPK3-silenced cells co-transfected with OE-STAT3. **P < 0.01, ***P < 0.001.

### 3.6 Silencing circHIPK3 attenuates B-ALL progression *in vivo*

To validate these findings *in vivo*, a B-ALL xenograft model was established using NOD-SCID mice. While control mice developed severe splenomegaly indicative of a high tumor burden, circHIPK3 silencing markedly reduced spleen size and weight (P = 0.0004; Figures 6A,B). The sh-circHIPK3 group exhibited a significantly reduction in circHIPK3 and STAT3 levels compared to the sh-NC group (P < 0.001; Figures 6C,D). H&E staining revealed that sh-NC spleens were heavily infiltrated by hyperchromatic leukemic cells, disrupting normal tissue architecture. In contrast, sh-circHIPK3 tissues maintained better structural integrity with sparse leukemic infiltration (Figure 6E). Flow cytometry analysis demonstrated a significant decrease in the percentage of human CD45^+^CD19^+^ cells in both the bone marrow and spleens of sh-circHIPK3 mice (P < 0.001; Figure 6F), confirming that circHIPK3 depletion impedes leukemic dissemination *in vivo*.

**FIGURE 6:**
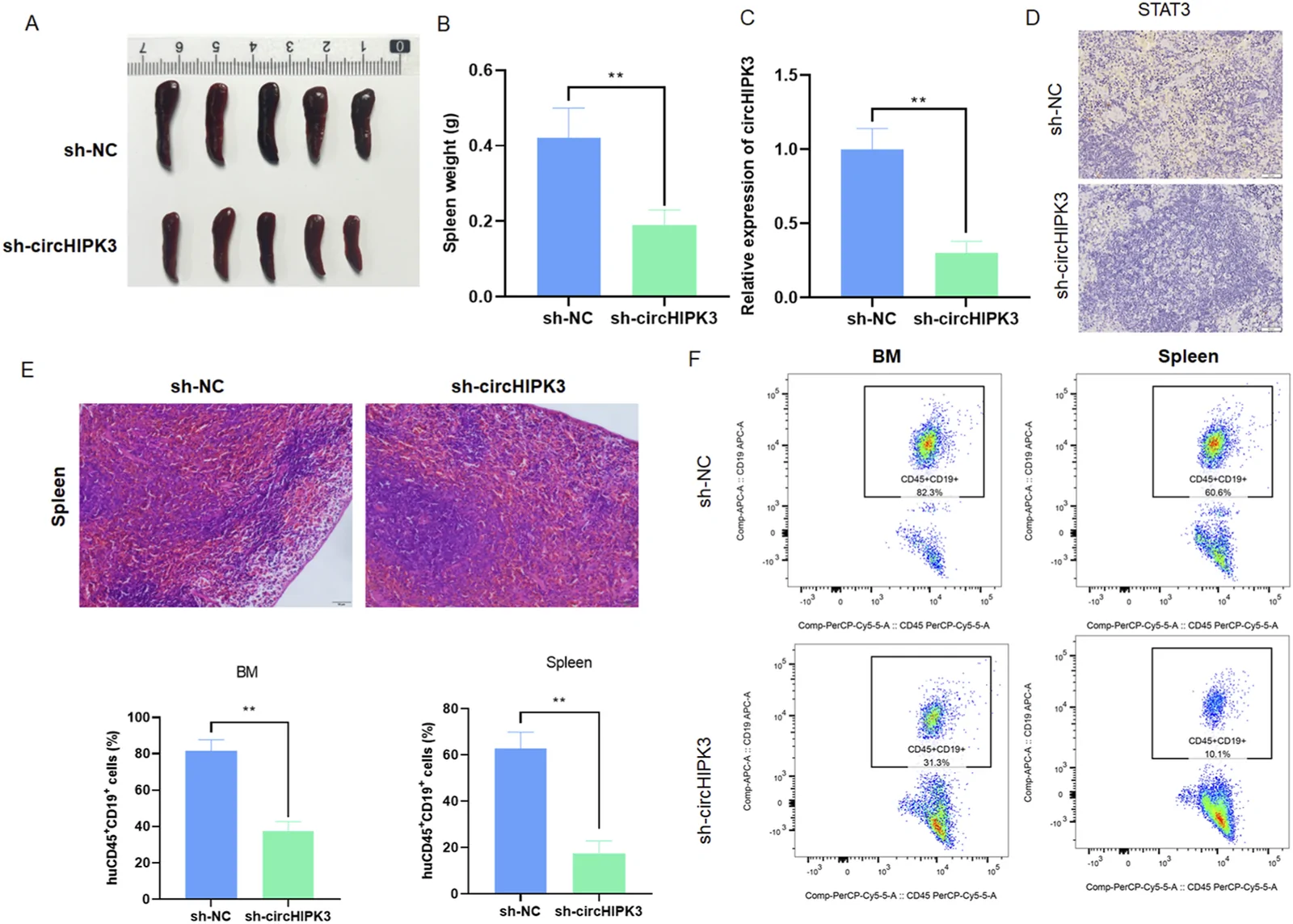
Silencing circHIPK3 impedes B-ALL progression *in vivo*. **(A)** Representative photographs of spleens harvested from the xenograft mice (n = 5). **(B)** Comparison of spleen weights between the sh-NC and sh-circHIPK3 groups (n = 5). **(C)** RT-qPCR analysis of circHIPK3 expression in spleen sections (n = 5). **(D)** Representative IHC images of STAT3 in spleen sections (n = 5). Scale bar = 50 μm. **(E)** Representative H&E staining images of spleen sections. Scale bar = 50 μm. **(F)** Flow cytometric analysis quantifying the percentage of human CD45^+^CD19^+^ leukemic cells in the bone marrow (BM) and spleens of the mice. **P < 0.01.

## 4 Discussion

The growing recognition of immune-mediated hematologic disorders associated with modern cancer therapies, including pembrolizumab-induced autoimmune hemolytic anemia, further emphasizes the need to better understand molecular regulatory pathways that govern hematologic cell survival and immune responses (Liu et al., 2026). The dysregulation of circRNAs has recently moved to the forefront of cancer research, revealing a sophisticated layer of post-transcriptional regulation that governs leukemogenesis. In the present study, we identified circHIPK3 as an oncogenic driver in pediatric B-ALL. Our data demonstrate that circHIPK3 is aberrantly upregulated in B-ALL and that its silencing effectively impairs leukemic progression both *in vitro* and *in vivo*. Mechanistically, we demonstrate a mode of action wherein cytoplasmic circHIPK3 acts as a molecular scaffold to association with STAT3, thereby shielding it from ubiquitin-mediated proteasomal degradation. This study not only highlights the therapeutic potential of targeting circHIPK3 but also expands our understanding of circRNA-protein interplay in hematological malignancies.

While circHIPK3 has been extensively studied in solid tumors, its role often varies depending on the cellular context. For instance, it functions as a tumor promoter in esophageal squamous cell carcinoma and hepatocellular carcinomas by acting as a “sponge” for miR-637 or miR-124 (Cao et al., 2024; Chen et al., 2018). In chronic myeloid leukemia, circHIPK3 has been identified as a potential biomarker (Gomez et al., 2024; Feng et al., 2020). Importantly, a previous study by Gaffo et al. comprehensively characterized circRNA expression in sorted B-cells, T-cells, and monocytes from healthy donors and identified circHIPK3 as one of the most significantly upregulated circRNAs in pediatric B-precursor ALL even when using sorted normal B-cells as the comparator ALL (Gaffo et al., 2019). Our findings in B-ALL align with the oncogenic profile observed in other aggressive cancers. CircHIPK3 knockdown prevented the malignant phenotype of B-ALL cells, manifested as suppressed proliferation and increased apoptosis. However, unlike the canonical “miRNA sponge” mechanism that dominates current literature, our study provides evidence for a protein-binding function. Ding et al. revealed that circHIPK3 acts as a scaffold to promote ubiquitin ligase-mediated degradation of HUR (Ding et al., 2022). Li et al. demonstrated that circHIPK3 can physically interact with DRP1, positively regulating its expression and thereby controlling necrotic apoptosis in vascular smooth muscle cells (Li et al., 2025). Furthermore, evidence indicates that circHIPK3 can bind to STAT3 in macrophages, thereby inhibiting STAT3 protein degradation (Cai et al., 2024). However, it remains unknown whether circHIPK3 regulates B-ALL by interacting with STAT3. Although we cannot entirely rule out the possibility that circHIPK3 may also sponge miRNAs in B-ALL, the rescue experiments involving STAT3 overexpression almost completely reversed the phenotype induced by circHIPK3 knockdown. This suggests that the modulation of STAT3 protein stability is a predominant mechanism driving circHIPK3-mediated leukemogenesis in this context.

STAT3 is a constitutively activated transcription factor in diverse malignancies, including B-ALL, where it drives cell survival and drug resistance (Gasparoli et al., 2024; Bhansali et al., 2021). While most studies focus on the phosphorylation status of STAT3 via upstream JAK kinases (Wang et al., 2024; Kuang et al., 2019), its protein abundance is equally regulated through the ubiquitin-proteasome system (UPS). Yang et al. uncovered that circFOXP1 binds to the STAT3 protein and inhibits its ubiquitination and subsequent degradation (Yang J. et al., 2023). In arsenic-induced bladder carcinogenesis, circPDE3B directly binds to STAT3, reducing STAT3 ubiquitination and increasing its stability (Gao et al., 2025). Our results indicated that circHIPK3 directly interacted with STAT3 protein and enhanced its stability by antagonizing ubiquitin-proteasome degradation. It is worth noting that while the interaction between circHIPK3 and STAT3 has been documented in other cancer types, our study reveals a fundamentally distinct regulatory mechanism. Previous reports predominantly described circHIPK3 as a competitive endogenous RNA (ceRNA) that sponges specific miRNAs (such as miR-124-3p, miR-637) to upregulate STAT3 mRNA levels (Chen et al., 2020; Hu and Zhang, 2019; Zhang et al., 2019). Another study revealed that circHIPK3 sequesters IGF2BP2 to regulate STAT3 mRNA stability (Okholm et al., 2024). However, our mechanistic investigations revealed that in B-ALL, circHIPK3 regulates STAT3 at the post-translational level rather than the transcriptional level, suggesting that STAT3 dysregulation in B-ALL occurs at multiple molecular levels. STAT3 upregulation likely reflects the combined effects of oncogenic transcriptional activation in B-ALL and potentially circHIPK3-mediated mRNA stabilization through ceRNA or IGF2BP2 sequestration mechanisms, as described in other cancer types (Yang J. et al., 2023; Gao et al., 2025; Chen et al., 2020; Hu and Zhang, 2019). Although a recent study reported physical interaction between circHIPK3 and STAT3 protein in macrophages (Cai et al., 2024), that work did not address whether this interaction affects STAT3 protein turnover. Our study fills this critical gap by establishing a complete functional cascade from direct protein binding to stabilization of functional STAT3 signaling. This finding is significant because it implies that disrupting the circHIPK3 and STAT3 complex could offer a strategy to degrade oncogenic STAT3, distinct from using kinase inhibitors.

The translational relevance of our findings is underscored by the *in vivo* xenograft experiments. Silencing circHIPK3 not only extended the overall survival of mice but also significantly reduced splenomegaly and leukemic infiltration in the bone marrow. High tumor burden and extramedullary infiltration are hallmarks of poor prognosis in B-ALL (Huang et al., 2025; Yang Q. et al., 2023; Zhu et al., 2023). The fact that circHIPK3 depletion could structurally restore splenic architecture suggests its potential as a target to limit leukemic dissemination. Furthermore, the high stability of circHIPK3, attributed to its circular structure, makes it an attractive candidate for a diagnostic or prognostic biomarker in clinical settings.

Despite these promising findings, our study has limitations that warrant future investigation. First, the clinical cohort is relatively small (n = 30 per group), which may limit statistical power for subgroup analyses; validation in larger, multi-center cohorts is warranted. Second, the use of bulk BMMCs/PBMCs from healthy donors as controls, rather than sorted normal B-cell precursors, represents a potential limitation, as differences in cell composition and proliferative status between leukemic blasts and normal mononuclear cells could theoretically contribute to the observed circHIPK3 expression differences. Future validation using sorted normal B-cell precursors would further strengthen this conclusion. Third, while we confirmed that circHIPK3 prevents STAT3 ubiquitination, the specific E3 ubiquitin ligase responsible for tagging STAT3 in B-ALL cells were not identified in this study. Future work should focus on identifying whether circHIPK3 disrupts the binding of known STAT3-targeting E3 ligases. Fourth, although we utilized a xenograft model, the use of patient-derived xenografts (PDX) in future studies would better recapitulate the heterogeneity of human B-ALL.

In conclusion, our study establishes circHIPK3 as a pivotal promoter of B-ALL progression. We characterize a specific mechanism whereby circHIPK3 stabilizes the STAT3 oncoprotein by antagonizing ubiquitin-proteasomal degradation. These insights provide a rationale for developing RNA-based therapeutics or small molecule inhibitors disrupting the circHIPK3-STAT3 complex as an approach to combat B-ALL.

## Data Availability

The original contributions presented in the study are included in the article/Supplementary Material, further inquiries can be directed to the corresponding author.
