# Supplementary material for: CircHIPK3 promotes the progression of B-cell acute lymphoblastic leukemia in children by binding to STAT3
Source: Front Pharmacol. 2026 Sep 17;17:1884978. doi: 10.3389/fphar.2026.1884978 (PMC13627337; doi:10.3389/fphar.2026.1884978)
Supplement: Supplementary file 1 [file Image1.pdf]

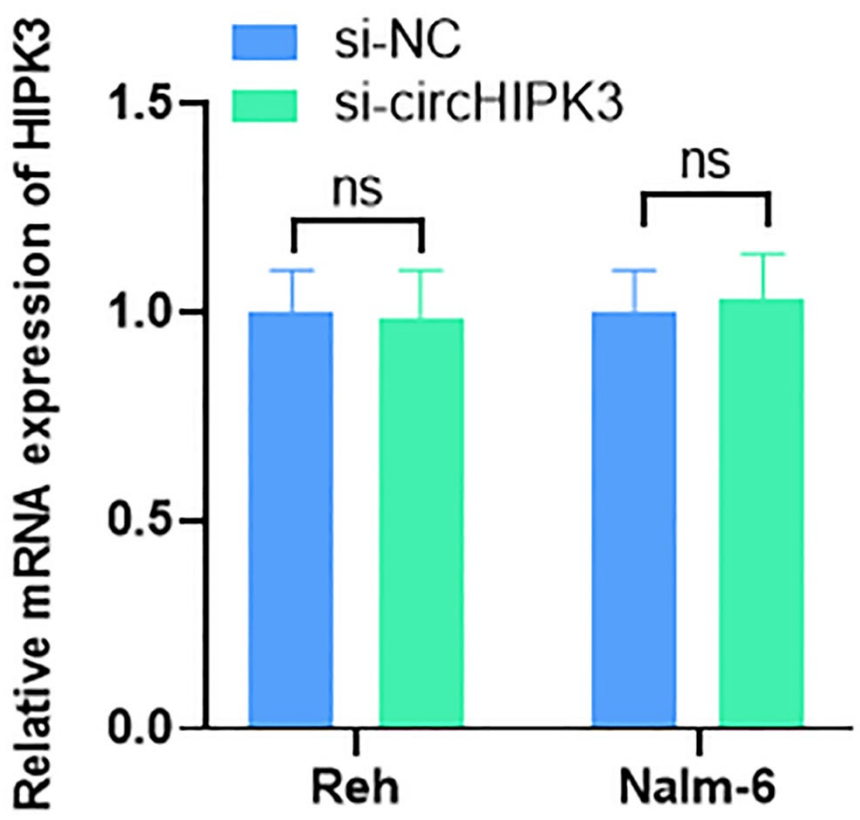

Figure S1 HIPK3 mRNA levels in Reh and Nalm-6 cells transfected with sh-NC or sh-circHIPK3 were measured by RT-qPCR.

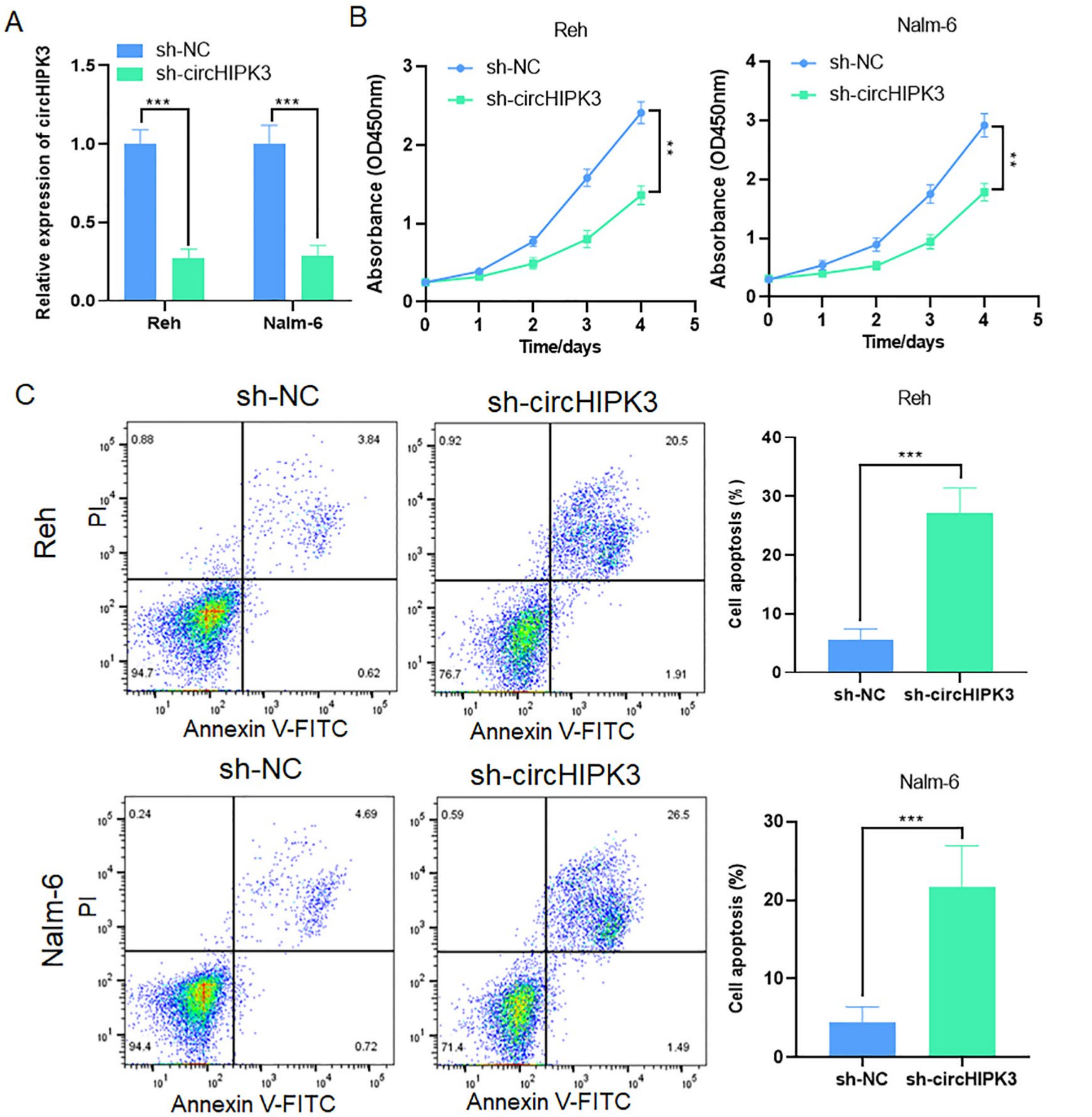

Figure S2 Depletion of circHIPK3 suppresses leukemic cell growth and induces cell cycle arrest and apoptosis. (A) CircHIPK3 levels in Reh and Nalm-6 cells transfected with sh-NC or sh-circHIPK3 were measured by RT-qPCR. (B) Assessment of cell viability using CCK-8 assays at 0, 1, 2, 3, and 4 day post-transfection. (C) Flow cytometry evaluation of apoptosis rate via Annexin V-FITC/PI double staining. ***P < 0.001.
