# Supplementary material for: CircHIPK3 promotes the progression of B-cell acute lymphoblastic leukemia in children by binding to STAT3
Source: Front Pharmacol. 2026 Sep 17;17:1884978. doi: 10.3389/fphar.2026.1884978 (PMC13627337; doi:10.3389/fphar.2026.1884978)
Supplement: Supplementary file 2 [file Supplementaryfile2.docx]

Figure 3

STAT3

GAPDH

Figure 4

STAT3

GAPDH

IB

FLAG-STAT3

FIGURE 5

STAT3

P-STAT3

BCL-2

c-Myc

GAPDH
