# Supplementary material for: CircHIPK3 promotes the progression of B-cell acute lymphoblastic leukemia in children by binding to STAT3
Source: Front Pharmacol. 2026 Sep 17;17:1884978. doi: 10.3389/fphar.2026.1884978 (PMC13627337; doi:10.3389/fphar.2026.1884978)
Supplement: Supplementary file 3 [file Supplementaryfile1.doc]

Supplementary Table 1

**Primer sequence**

circHIPK3	Forward 5'-TATGTTGGTGGATCCTGTTCGGC-3'

Reverse 5'-TGGTGGGTAGACCAAGACTTGTGA-3'

HIPK3 mRNA	Forward 5'-TGGAGACTGGGGGAAGATGA-3'

Reverse 5'-CACACTAACTGGCTGAGGGG-3'

STAT3 mRNA	Forward 5'-CAGCAGCTTGACACACGGTA-3'

Reverse 5'-CACACTAACTGGCTGAGGGG-3'

GAPDH mRNA	Forward 5'-AAACACCAAAGTGGCATGTGA-3'

Reverse 5'-GCTTCACCACCTTCTTGATG-3'

**siRNA sequence**

circHIPK3 siRNA	sense 5'-GGUACUACAGGUAUGGCCUTT-3'

antisense 5'-AGGCCAUACCUGUAGUACCTT-3'

**shRNA** **target sequence**

5'-GATCCGCTACTACAGGTATGGCCTCACAATTCAAGAGATTGTGAGGCCATACCTGTAGTAGTTTTTTA-3'

**FISH probe**

circHIPK3	5'-TGTGAGGCCATACCTGTACCGAGATTG-3'

**RNA pulldown**

circHIPK3 Anti-sense probe 5'-CAATCTCGGTACTACAGGTATGGCCTCACAAGTC-3'

circHIPK3 sense probe	5'-GACTTGTGAGGCCATACCTGTAGTACCGAGATTG-3'
